# Interpretable machine learning model integrating CT radiomics, CTR, and clinical features for EGFR mutation prediction in ≤3 cm lung adenocarcinoma nodules

**DOI:** 10.1080/07853890.2025.2607160

**Published:** 2025-12-19

**Authors:** Wenhan Cai, Yiming Liu, Kai Zhao, Zirui Zhu, Jiamei Jin, Herui Han, Mingchuan Hu, Xiangming Qiu, Jiaxin Wen, Zhiqiang Xue

**Affiliations:** a Graduate School, Chinese PLA General Hospital, Beijing, China; b Department of Thoracic Surgery, First Medical Center, Chinese PLA General Hospital, Beijing, China; c Department of Thoracic Surgery, Hainan Hospital, Chinese PLA General Hospital, Sanya, China; d School of Medicine, Nankai University, Tianjin, China

**Keywords:** Lung adenocarcinoma, EGFR mutation, radiomics, consolidation-to-tumor ratio, CT imaging, machine learning, SHAP

## Abstract

**Background:**

Non-invasive prediction of EGFR mutation status in lung adenocarcinoma (LUAD) is critical for treatment planning, particularly in small pulmonary nodules where tissue genotyping is limited. However, the consolidation-to-tumor ratio (CTR), a clinically relevant imaging biomarker, has rarely been incorporated into radiomics-based models.

**Objective:**

To develop and validate an interpretable CT radiomics model incorporating CTR and clinical features for predicting EGFR mutation status in LUAD patients with nodules ≤3 cm.

**Methods:**

In this retrospective study included 492 patients with pathologically confirmed LUAD who underwent preoperative non-contrast chest CT between January 2017 and December 2022. Tumors were manually segmented for radiomic feature extraction, and CTR was measured for each lesion. Radiomic textures were computed with PyRadiomics using a fixed gray-level bin width. Feature selection was performed using analysis of variance and mutual information filtering followed by RFE with a random-forest base estimator. Three random forest classifiers were constructed: a radiomics-only model, a clinical-only model, and a combined radiomics-clinical model. Model performance was assessed by AUC with 95% CI, and interpretability was evaluated using SHapley Additive exPlanations (SHAP).

**Results:**

The combined model achieved the best performance (AUC, 0.74 [95% CI: 0.69–0.79] in training; 0.76 [95% CI: 0.66–0.85] in testing), outperforming the radiomics-only (AUC, 0.69) and clinical-only (AUC, 0.60) models in the testing cohort. CTR was the most influential feature according to SHAP analysis.

**Conclusion:**

A interpretable radiomics model integrating CTR and clinical features enables effective non-invasive prediction of EGFR mutation status in small LUAD nodules, supporting molecular risk stratification when tissue genotyping is unavailable.

## Introduction

Lung adenocarcinoma (LUAD), the most common subtype of lung cancer, accounts for approximately 40% of all cases and remains a leading cause of cancer-related mortality worldwide. In 2022 alone, approximately 2.5 million new cases of lung cancer were diagnosed globally [1]. Among LUAD patients, epidermal growth factor receptor (EGFR) mutations are present in 40%–50% of Asian patients and 10%–20% of Caucasian patients, significantly influencing treatment decisions [2]. EGFR-tyrosine kinase inhibitors (EGFR-TKIs) have transformed the treatment landscape, improving progression-free survival (PFS) from 4–6 months with chemotherapy to 9–12 months with first-generation TKIs, while third-generation TKIs, such as osimertinib, have further extended overall survival (OS) to approximately 38 months in patients with advanced EGFR-mutant LUAD [3]. These data underscore the critical need for accurate EGFR mutation detection to optimize patient outcomes.

Currently, EGFR mutation status is determined *via* tissue biopsy, which remains the gold standard but has several inherent limitations [4]. First, biopsy is invasive and carries procedural risks, including bleeding, infection, and pneumothorax, particularly in patients with poor pulmonary function or comorbidities [5]. Second, obtaining sufficient tumor tissue for molecular testing can be challenging, especially in small or difficult-to-access lesions, leading to a high rate of inconclusive results [6,7]. Third, tissue biopsies capture only a snapshot of the tumor, potentially missing subclonal EGFR mutations due to tumor heterogeneity [8,9]. Additionally, the high cost and turnaround time of molecular testing pose barriers to widespread implementation, particularly in resource-limited settings [10–12]. These challenges highlight the need for a non-invasive, efficient, and comprehensive method to predict EGFR mutation status in LUAD patients.

Radiomics, an emerging approach that extracts high-dimensional quantitative features from medical imaging, has shown promise in predicting genetic alterations in tumors [13,14]. By analyzing texture, shape, and intensity patterns, radiomics enables non-invasive tumor characterization and may complement or replace biopsy-based genetic testing [15]. Several studies have reported high predictive performance using radiomics-based models for EGFR mutation prediction [16–18]. However, many existing models treat LUAD nodules as a homogeneous group and overlook visually accessible imaging characteristics that may carry clinical significance. Semantic features such as consolidation-to-tumor ratio (CTR), lobulation, and pleural retraction are routinely observed in CT scans and have been linked to tumor invasiveness and adverse prognosis [19]. CTR, in particular, provides a reproducible measure of the proportion of solid components within a pulmonary nodule and has been increasingly recognized as a relevant indicator in clinical assessment [20]. Despite this, CTR is rarely incorporated as a structured variable in radiomics-based models, limiting both interpretability and clinical integration.

To address this limitation, the present study aimed to develop a CT-based radiomics machine-learning model for predicting EGFR mutation status in LUAD patients with small pulmonary nodules (≤ 3 cm). The model was designed to integrate the CTR with radiomic features and relevant clinical variables, and to incorporate interpretability analysis to identify key imaging and clinical predictors. This approach is intended to provide a non-invasive, clinically applicable tool for molecular risk stratification in scenarios where tissue genotyping is unavailable or impractical.

## Materials and methods

### Patients

A retrospective search of the medical records at the First Medical Center of Chinese PLA General Hospital identified 893 patients with pathologically confirmed LUAD who underwent preoperative thin-slice chest CT (1–1.25 mm) between January 2017 and December 2022. Ethical approval was obtained from the Ethics Committee of Chinese PLA General Hospital (Approval No. S2024-377-01), and the requirement for informed consent was waived due to the retrospective study design.

Patients were eligible if they met the following inclusion criteria: (1) pathologically confirmed primary LUAD, (2) EGFR mutation status determined *via* tissue-based genetic testing, (3) availability of preoperative non-contrast thin-slice chest CT (slice thickness 1 mm or 1.25 mm), and (4) complete clinical and imaging data.

**Figure 1.**
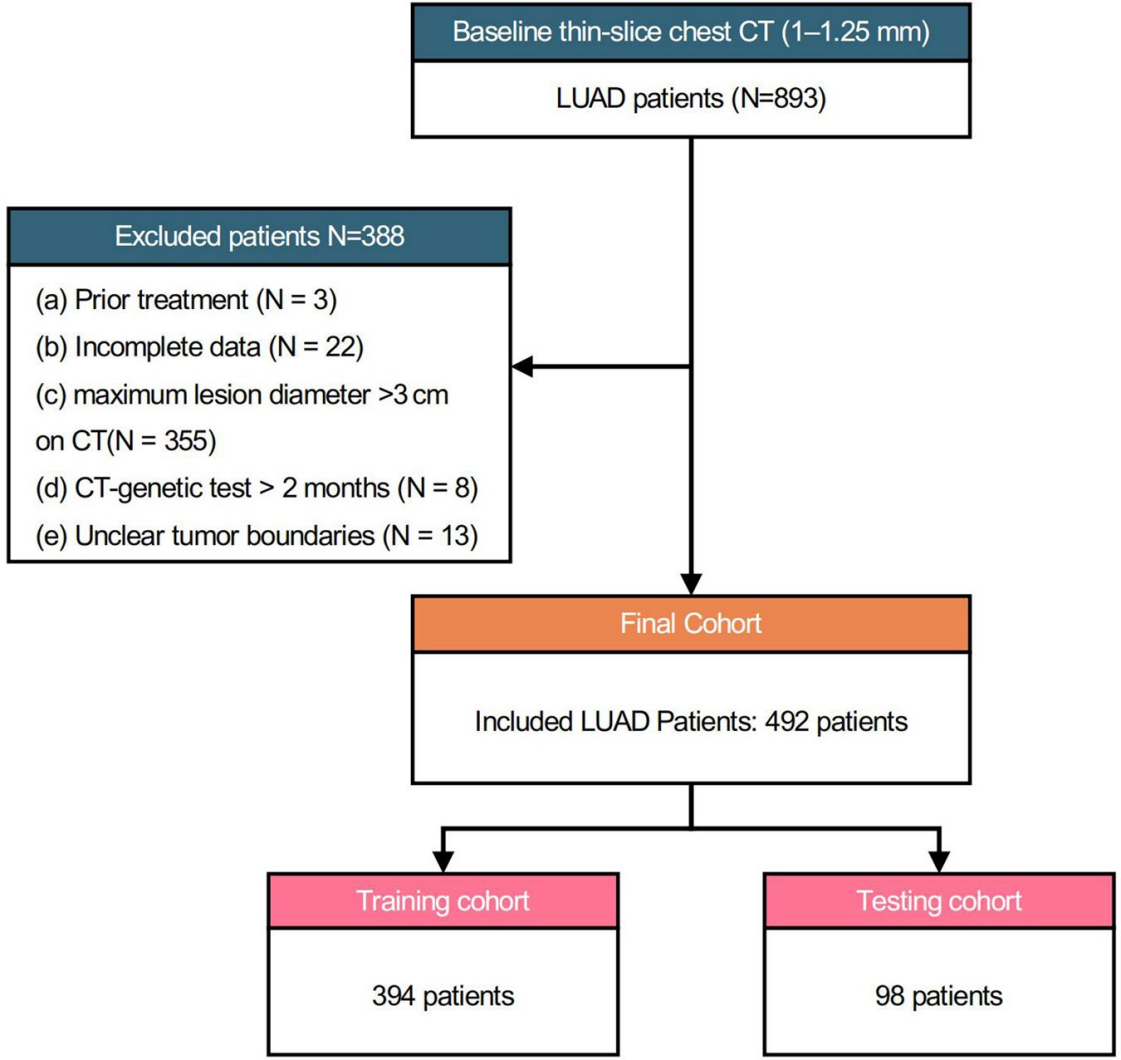
Patient selection flowchart. A total of 893 patients with LUAD who underwent baseline thin-slice chest CT (1–1.25 mm) were initially screened. After excluding patients with prior treatment (*n* = 3), incomplete data (*n* = 22), lesions >3 cm in maximum diameter on CT (*n* = 355), a time interval between CT and genetic testing exceeding 2 months (*n* = 8), or unclear tumor boundaries (*n* = 13), 492 patients were included in the final cohort. These patients were randomly divided into a training cohort (*n* = 394) and a testing cohort (*n* = 98).

Exclusion criteria were as follows: (a) prior anti-tumor treatment before surgery (*n* = 3); (b) incomplete clinical or imaging data (*n* = 22); (c) maximum lesion diameter >3 cm on CT (*n* = 355); (d) time interval between CT scan and EGFR testing >2 months (*n* = 8); and (e) tumors with ill-defined boundaries that precluded accurate region of interest (ROI) segmentation (*n* = 13) (Figure 1).

**Figure 2.**
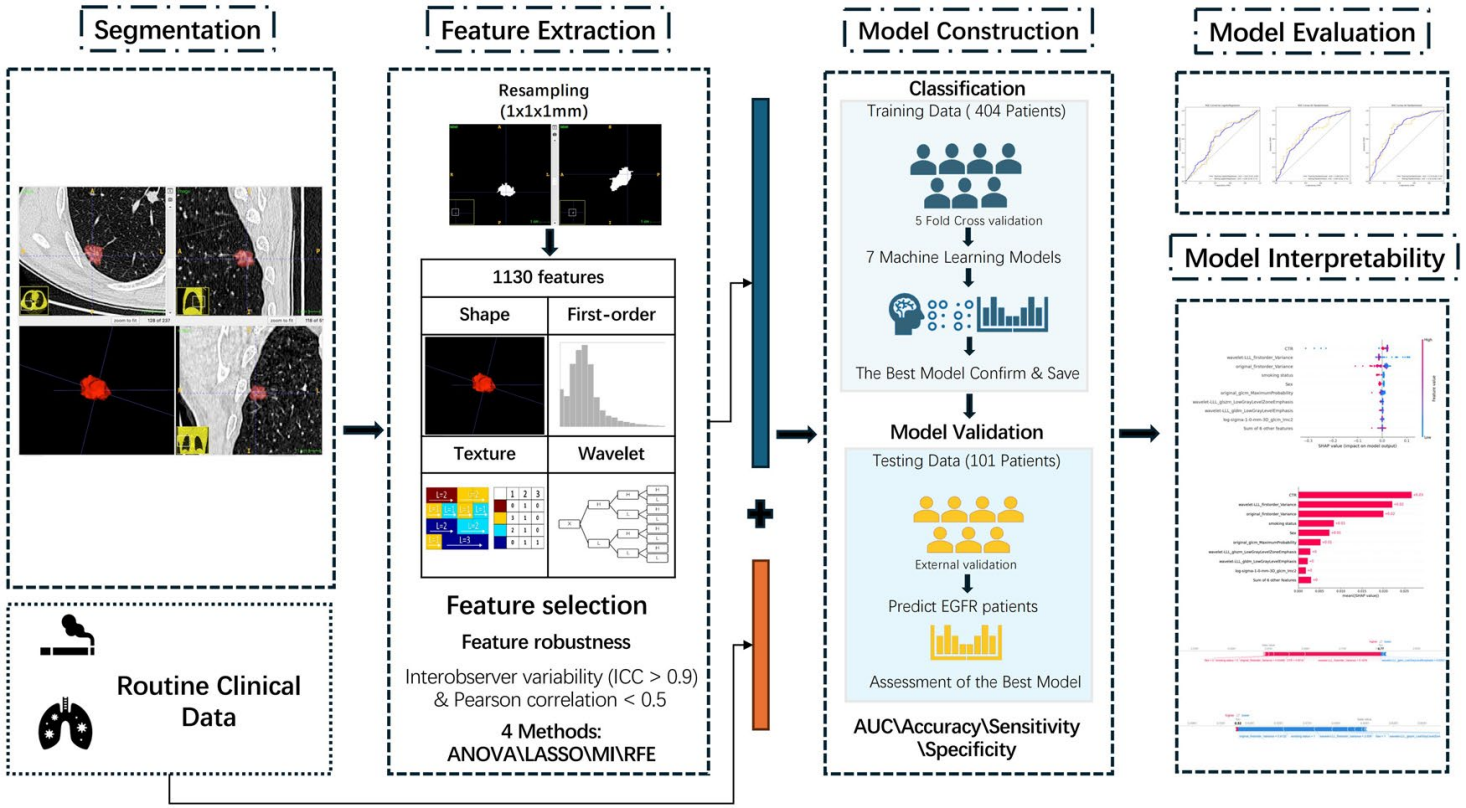
Workflow of the radiomics-based machine-learning model. (1) Segmentation of thin-slice CT; (2) Extraction of 1,130 radiomic features from resampled images (1 × 1 × 1 mm³); (3) Robustness filtering (ICC > 0.90), redundancy pruning *via* hierarchical clustering on absolute Spearman correlations (|ρ| ≥ 0.85), and univariate screening (ANOVA/MI), followed by RFE with a random-forest base estimator; (4) Model construction with five-fold cross-validation; (5) Evaluation in an independent test cohort; (6) Interpretability with SHAP.

After applying the above criteria, 492 patients were ultimately included. All enrolled cases had pulmonary nodules with a maximum diameter ≤3 cm and underwent successful ROI segmentation. The dataset was randomly divided into a training cohort (*n* = 394, 80%) and a testing cohort (*n* = 98, 20%). Stratified random sampling was performed to ensure balanced EGFR mutation status distribution between training and testing cohorts. The overall workflow is presented in Figure 2. The CTR was defined as the ratio of the maximum diameter of consolidation to the maximum tumor diameter, both measured on lung window settings of preoperative CT images. All measurements were performed along the same axis on axial slices. This definition was adapted from previously published studies [21,22]. To ensure methodological reproducibility, CTR was independently assessed by two experienced thoracic radiologists, with discrepancies resolved by consensus.

### EGFR mutation detection

EGFR mutation status was determined using tumor specimens obtained during surgical resection. Exons 18–21 of the EGFR gene, which harbor the most common activating mutations in LUAD, were analyzed using next-generation sequencing (NGS) following standardized clinical protocols. Patients with any mutation detected in these exons were classified as EGFR mutation-positive, while those without detectable mutations were categorized as EGFR wild-type.

### CT image acquisition, preprocessing, and ROI segmentation

All enrolled patients underwent non-contrast chest CT scans using a 256-slice CT scanner (United Imaging, China). Scanning parameters varied depending on the detector configuration: for 64-detector scans, 110 kVp with tube current modulation and a pitch of 0.85; for 128-detector scans, 120 kVp with a pitch of 1.2; and for 256-detector scans, 100 kVp with a pitch of 0.992. All images were reconstructed with a slice thickness and interval of 1.0–1.5 mm using a standard smoothing kernel, and exported in DICOM format.

In-plane pixel spacing ranged from 0.5 to 1.1 mm, while slice spacing ranged from 1.0 to 1.5 mm. To standardize spatial resolution, all CT volumes were resampled to isotropic voxels of 1 × 1 × 1 mm³ using linear interpolation.

Tumor regions of interest (ROIs) were manually delineated on axial CT images by three board-certified thoracic radiologists (each >5 years’ experience) using ITK‑SNAP, contouring the entire lesion volume slice-by-slice. All segmentations underwent visual quality control. Interobserver agreement was evaluated in 30 randomly selected cases independently segmented by two radiologists; the mean Dice similarity coefficient (DSC) was 0.91 ± 0.03, indicating excellent reproducibility. These verified segmentations were used for radiomic feature extraction.

### Radiomic feature extraction and selection

Radiomic features were extracted with PyRadiomics (v3.0.1) following Image Biomarker Standardisation Initiative (IBSI) guidelines. Prior to texture calculation, voxel intensities were discretized using a fixed bin width (binWidth = 25) to harmonize gray-level distributions. A total of 1130 features were initially extracted from CT images, including shape features, first-order statistics, texture features derived from gray-level co-occurrence matrix (GLCM), gray-level run length matrix (GLRLM), gray-level size zone matrix (GLSZM), dependence matrices (GLDM) and wavelet-transformed features.

To ensure robustness, feature-wise interobserver reliability was assessed in the same 30-case subset with duplicate segmentations using a two-way random-effects, absolute-agreement ICC; features with ICC < 0.90 were excluded. Remaining features were z-score normalized. Redundancy was addressed using hierarchical clustering on the absolute Spearman correlation matrix among features. A correlation threshold (e.g. |ρ| ≥ 0.85) defined clusters; from each cluster, one representative feature was retained based on the strongest univariate association with EGFR status. Multiple feature selection methods—analysis of variance (ANOVA), mutual information (MI), least absolute shrinkage and selection operator (LASSO), and recursive feature elimination (RFE)—were compared to identify the most discriminative subset. Among these, a RFE procedure with a random forest classifier as the base estimator was then used to select the final multivariate subset.

### Radiomics model development and performance evaluation

After radiomic feature extraction and selection, we constructed the radiomics model using seven machine learning algorithms: support vector machine (SVM), logistic regression, k-nearest neighbors (KNN), decision tree, random forest, XGBoost, and LightGBM. All models were trained on the training cohort using five-fold cross-validation and evaluated in the independent testing cohort. Hyperparameter optimization was performed *via* grid search based on the average area under the ROC curve (AUC). Among these algorithms, the random forest model demonstrated the most robust and consistent performance and was therefore selected as the final algorithm for subsequent model evaluation. Model performance was assessed using ROC curves and AUC values.

### Clinical and combined model construction

To explore the added value of clinical features, a separate clinical prediction model was established. Univariate statistical tests were first conducted within the training cohort to identify EGFR-associated clinical variables. Variables with significant differences between EGFR-positive and EGFR-negative patients (*p* < 0.05), including sex, smoking history, and CTR, were selected for model construction. A logistic regression model was developed based on these clinical predictors.

Subsequently, to evaluate the complementary value of radiomics and clinical information, a combined model was constructed by integrating the selected clinical variables with the RFE‑selected radiomics subset features. This integrated model was developed using the same training and validation strategy as the radiomics model, with random forest as the classifier. For benchmarking, radiomics‑only and clinical‑only pipelines were built analogously in both the training and testing cohorts to assess their relative predictive performance.

### Model interpretability

Model interpretability was assessed using SHapley Additive exPlanations (SHAP). SHAP analysis was applied to the final random forest model to quantify the contribution of individual features to EGFR mutation prediction. SHAP analysis was applied to the final random forest model to quantify the contribution of individual features to EGFR mutation prediction. SHAP values were computed using TreeExplainer from the SHAP Python package, and summary plots and force plots were generated to visualize feature contributions. SHAP summary plots were generated to visualize the most influential features, providing insights into how specific radiomic attributes influenced model decisions. These interpretability analyses enhanced transparency and supported the clinical plausibility of the radiomics-based prediction framework.

### Statistical analysis

All statistical analyses were performed using R (version 4.2.1) and Python (version 3.12.10). Continuous variables were expressed as means ± standard deviations, while categorical variables were summarized as counts and percentages. Group comparisons between EGFR-mutant and EGFR-wild-type patients were conducted using independent-samples t-tests or Mann–Whitney U tests for continuous variables, and chi-square tests or Fisher’s exact tests for categorical variables. A two-tailed P value < 0.05 was considered statistically significant. Model performance was primarily evaluated using the AUC with 95% confidence intervals. In addition, threshold-dependent performance metrics, including accuracy, sensitivity, specificity, positive predictive value (PPV), and negative predictive value (NPV), were calculated to provide a comprehensive assessment across radiomics, clinical, and combined models. Model calibration was further assessed using calibration plots (decile-based calibration curves), calibration intercepts and slopes, and Brier scores (including scaled Brier scores), to evaluate the agreement between predicted probabilities and observed outcomes. Clinical utility of the models was evaluated through decision curve analysis (DCA), which quantified the net benefit across a range of clinically relevant threshold probabilities by comparing each model against treat-all and treat-none strategies. DeLong’s test was used to compare AUCs between the three models (radiomics-only, clinical-only, and combined) in the independent testing cohort, providing pairwise, non-parametric comparisons of ROC curves. Model robustness was assessed using the bootstrap method with 1,000 iterations and resampling equal to the original dataset size. All visualizations and statistical analyses were performed using R packages including ‘ggplot2’, ‘pROC’, ‘ggstatsplot’, and ‘ggcor’ as well as Python packages such as SciPy, scikit-learn, glmnet, caret, and SHAP.

## Results

### Clinical characteristics of patients

The clinical characteristics of the 493 patients with pathologically confirmed LUAD are summarized in Table 1. A total of 394 patients were assigned to the training cohort (EGFR-positive: 231; EGFR-negative: 163), and 98 patients were assigned to the testing cohort (EGFR-positive: 57; EGFR-negative: 41). In the training cohort, EGFR-mutant patients had a significantly higher proportion of females (64.50% vs. 53.37%, *p* = 0.0264), more non-smokers (77.92% vs. 67.48%, *p* = 0.0206), and larger CTR (0.66 ± 0.02 vs. 0.57 ± 0.03, *p* = 0.0071) compared with EGFR wild-type patients. No significant differences were observed in age, lobulation, vacuole sign, pleural retraction, tumor family history, or TNM staging between the two groups.

**Table 1.** Clinical characteristics of enrolled patients.

	Training cohort		p value	Testing cohort		p value
	EGFR(-)	EGFR(+)		EGFR(-)	EGFR(+)	
counts	163	231		41	57	
Age	56.51 ± 0.85	56.42 ± 0.66	0.9361	56.95 ± 1.79	54.17 ± 1.18	0.1991
Sex			0.0264			0.0221
Male	76(46.63)	82(35.50)		20(48.78)	15(26.32)	
Female	87(53.37)	149(64.50)		21(51.22)	42(73.68)	
Smoking history			0.0206			0.0663
No	110(67.48)	180(77.92)		27(65.85)	46(82.14)	
Yes	53(32.52)	51(22.08)		14(34.15)	10(17.86)	
CTR	0.57 ± 0.03	0.66 ± 0.02	0.0071	0.60 ± 0.06	0.73 ± 0.03	0.0489
Vacuole			0.0723			0.6252
No	144(88.34)	216(93.51)		37(90.24)	53(92.89)	
Yes	19(11.66)	15(6.49)		4(9.76)	4(7.02)	
Lobulation			0.318			0.1135
No	140(85.89)	207(89.22)		34(83.33)	54(94.92)	
Yes	23(14.11)	25(10.78)		7(16.67)	3(5.08)	
Pleural retraction			0.0846			0.8655
No	77(47.24)	89(38.53)		13(31.71)	19(33.33)	
Yes	86(52.76)	142(61.47)		28(68.29)	38(66.67)	
Tumor family history			0.0826			0.6478
No	116(71.17)	182(78.79)		30(73.17)	44(76.19)	
Yes	47(28.83)	49(21.21)		11(26.83)	13(23.81)	
T stage			0.2379			0.3598
0	2(1.23)	0(0.00)		1(2.44)	0(0.00)	
1	132(80.98)	188(81.39)		31(75.61)	40(70.18)	
2	29(17.79)	43(18.61)		9(21.95)	17(29.83)	
N stage			0.1943			0.7162
0	146(90.68)	222(95.28)		38(95.00)	55(96.49)	
1	12(7.45)	9(3.86)		2(5.00)	2(3.51)	
2	3(1.86)	2(0.86)		0(0.00)	0(0.00)	

In the testing cohort, a significantly higher proportion of EGFR-mutant patients were female (73.68% vs. 51.22%, *p* = 0.0221), and CTR values were higher in the EGFR-mutant group (0.73 ± 0.03 vs. 0.60 ± 0.06, *p* = 0.0489). Other baseline features including age, smoking history, lobulation, and tumor staging showed no significant intergroup differences.

### Feature selection for radiomic modeling

A total of 1130 radiomic features were initially extracted, encompassing shape, first-order, texture (GLCM, GLRLM, GLSZM), and wavelet-transformed features. After reproducibility filtering (ICC > 0.90), 912 robust features were retained. Multiple feature selection methods, including ANOVA, LASSO, MI, and RFE, were evaluated. Among them, the RFE achieved optimal performance in model validation and was therefore selected as the final method for identifying discriminative features. A total of 12 features were ultimately selected for radiomics model construction (details provided in Supplementary Table S1).

### Performance of clinical, radiomics, and combined models

**Figure 3.**
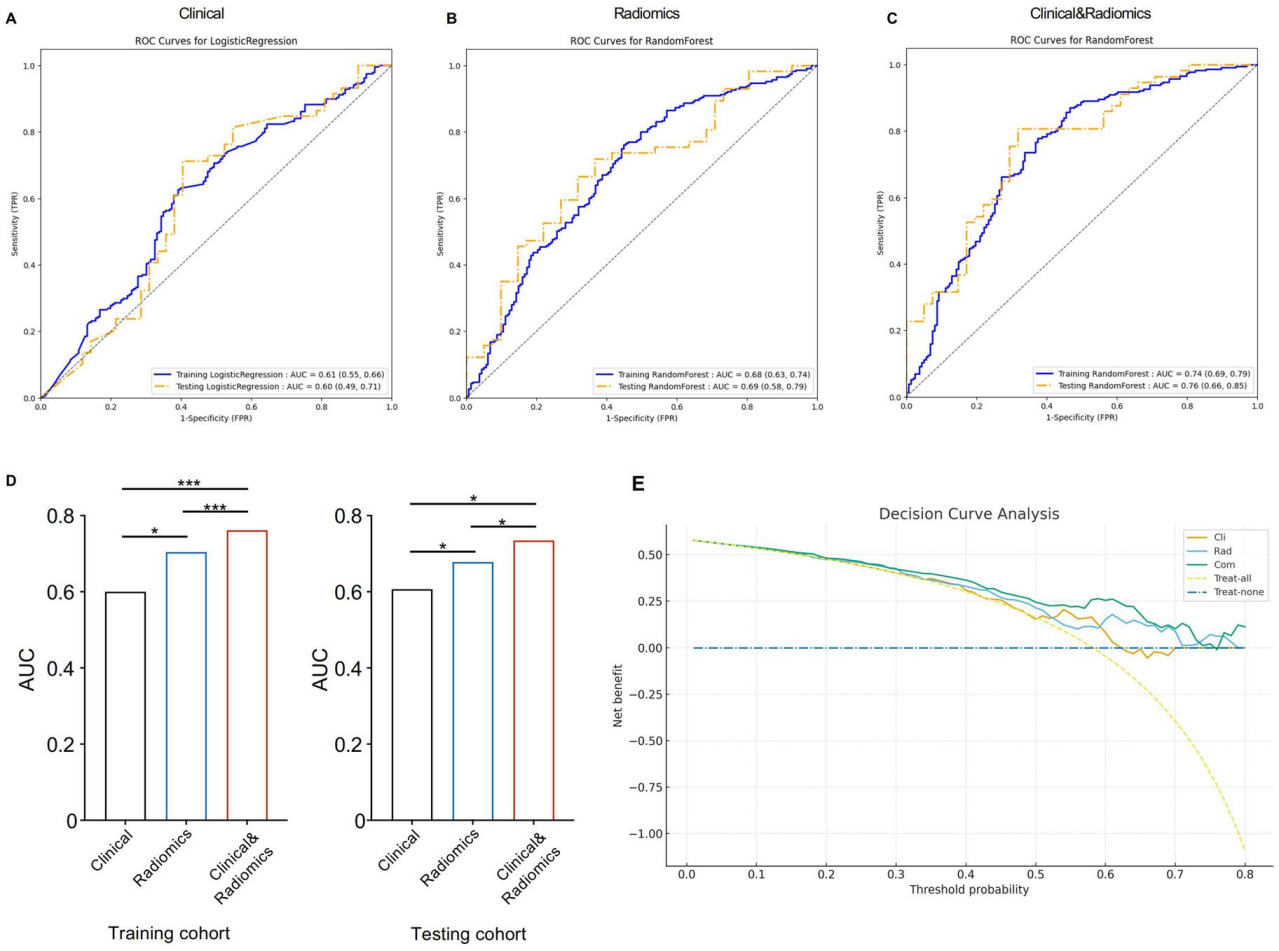
Performance comparison of clinical, radiomics, and combined models for EGFR mutation prediction. (A–C) ROC curves of the three models in the training and independent testing cohorts: (A) clinical model using logistic regression; (B) radiomics model using random forest; (C) combined model integrating clinical and radiomics features using random forest. (D) Bar plots showing AUC values of each model in the training and testing cohorts; statistical significance was assessed using DeLong’s test (*p < 0.05; ***p < 0.001). (E) Decision curve analysis (DCA) comparing the net benefit of the clinical-only (Cli), radiomics-only (Rad), and combined (Com) models across a range of threshold probabilities, with ‘treat-all’ and ‘treat-none’ strategies shown as references.

Among the machine learning algorithms evaluated, random forest demonstrated the best performance and was therefore selected for model construction. Due to the statistically significant differences in smoking history, sex, and CTR between EGFR-mutant and wild-type groups in the training cohort, these clinical variables were incorporated into the clinical model. The clinical model, built using sex, smoking history, and CTR, achieved an AUC of 0.61 (95% CI: 0.55–0.66) in the training cohort and 0.60 (95% CI: 0.49–0.71) in the test cohort (Figure 3A, Supplementary Table S2). The radiomics model, constructed using 12 features selected *via* RFE, yielded AUCs of 0.68 (95% CI: 0.63–0.74) and 0.69 (95% CI: 0.58–0.79) in the training and testing sets, respectively (Figure 3B, Supplementary Table S3). The combined model that incorporated both clinical and radiomic features achieved the best overall performance, with AUCs of 0.74 (95% CI: 0.69–0.79) in the training cohort and 0.76 (95% CI: 0.66–0.85) in the testing cohort (Figure 3C, Supplementary Table S4). In pairwise comparisons using DeLong’s test, the combined model demonstrated significantly higher AUCs than both the clinical model (training: *p* < 0.001; testing: *p* < 0.05) and the radiomics model (training: *p* < 0.001; testing: *p* < 0.05). These results indicate that the integration of radiomic and clinical features significantly improves predictive performance across both cohorts (Figure 3D). In addition, calibration plots and Brier scores (Supplementary Fig. S1, Supplementary Table S5) showed that the combined model achieved the best probability calibration and the lowest Brier score, indicating improved reliability compared with either single-source model. Moreover, DCA (Figure 3E) demonstrated that both the radiomics and clinical models provided net clinical benefit compared with the treat-all and treat-none strategies, while the combined model consistently achieved the highest net benefit across the clinically relevant threshold range (0.20–0.60), highlighting its potential for clinical applicability.

### Model interpretability via SHAP analysis

**Figure 4.**
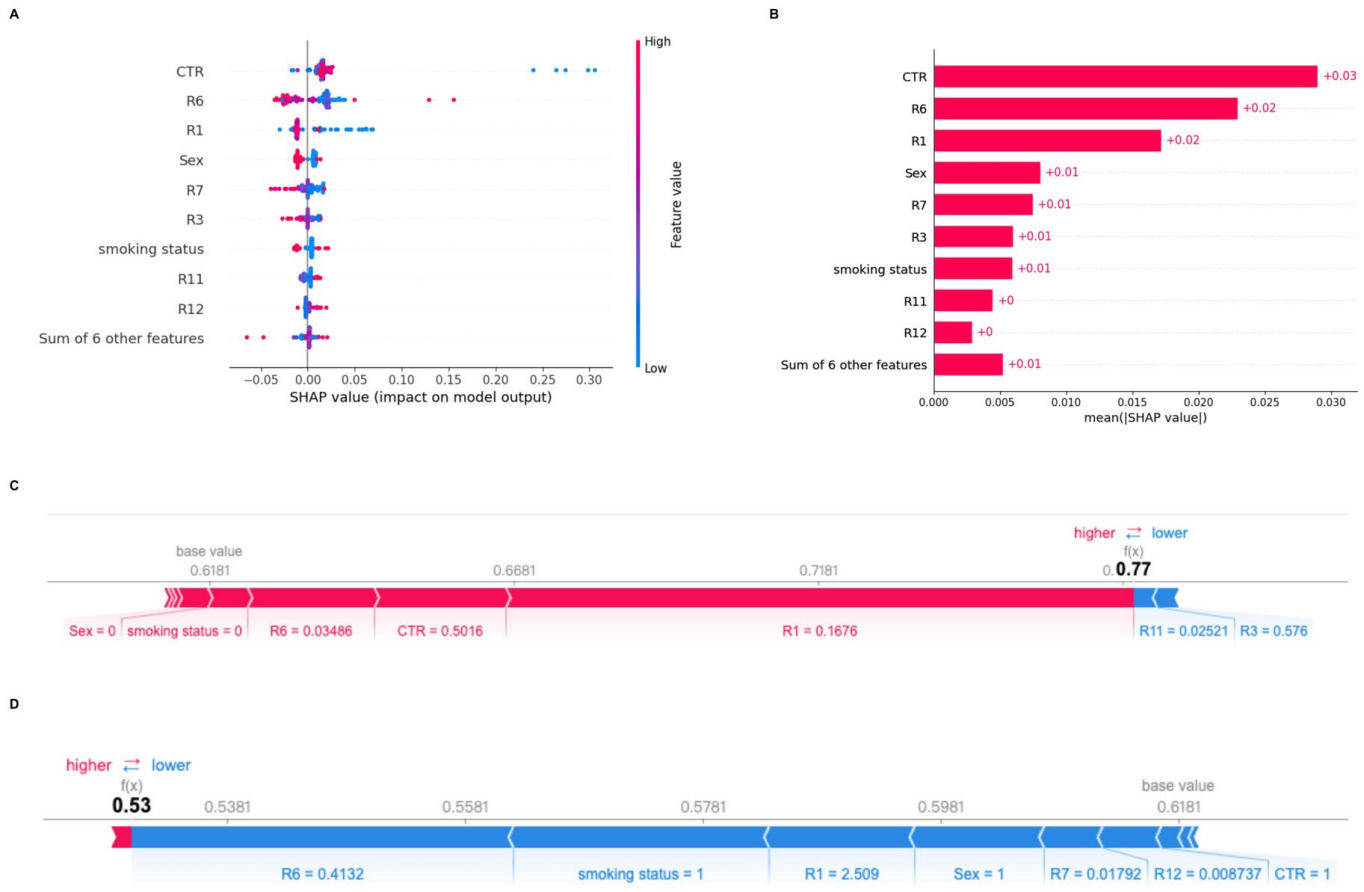
SHAP analysis for model interpretability. (A) SHAP summary plot showing the impact of each feature on model output in individual patients, where color represents feature value and horizontal position indicates positive or negative contribution. (B) Mean SHAP value ranking the overall feature importance. (C, D) SHAP force plots illustrating model output for representative patients with positive and negative EGFR mutation predictions. Red and blue bars denote features that push the prediction toward higher or lower risk, respectively. CTR, R6, and R1 were among the most influential predictors.

To improve the interpretability of the optimal random forest model, SHAP analysis was performed. The SHAP summary plot (Figure 4A) revealed that CTR had the greatest influence on EGFR mutation prediction, followed by radiomic features such as R1, R6, and clinical variables, including smoking status and sex. Higher CTR and greater variance values from first-order features were positively associated with EGFR mutation probability, while lower values of wavelet-transformed texture features (e.g. R12) contributed to wild-type classification.

The SHAP bar plot (Figure 4B) further quantified the average contribution of each feature, confirming CTR as the most impactful variable (mean SHAP value: +0.03), followed by R1 and R6 (both +0.02). SHAP force plots (Figure 4C, D) provided patient-level interpretations, illustrating how individual features influenced the model output toward either EGFR-mutant or wild-type status. Collectively, these findings highlight the complementary role of clinical and radiomic features in model decision-making and enhance its potential for transparent clinical application.

## Discussion

EGFR mutation status is a pivotal biomarker in LUAD, guiding treatment decisions and significantly improving outcomes through the use of TKIs [23,24]. However, EGFR mutation determination still primarily relies on tissue-based testing, which is invasive, costly, and often limited by sampling insufficiency or tumor heterogeneity [4,25,26]. These limitations are particularly prominent in patients with small-size nodules (≤3 cm), who may not routinely undergo genetic testing due to low perceived recurrence risk or the absence of indications for postoperative adjuvant therapy.

There is clinical value in developing noninvasive and interpretable tools for predicting mutation status in LUAD, particularly for small pulmonary nodules (≤ 3 cm) in which tissue genotyping is often unavailable or infeasible. The aim of our study was to establish and validate a CT radiomics–based machine learning model that integrates the CTR and clinical variables to predict EGFR mutation status. In this retrospective analysis, CTR, patient age, and selected tumor-related radiomic features were identified as key predictors through a combined feature selection strategy. These predictors were incorporated into a combined radiomics-clinical model, which achieved higher discriminative performance than either a radiomics-only model or a clinical-only model (AUC: 0.76 vs 0.69 and 0.60, respectively, in the testing set). Model interpretability analysis confirmed the dominant contribution of CTR and highlighted the complementary value of radiomic features in improving prediction accuracy across different patient subgroups.

Recent evidence suggests that EGFR mutations are associated with higher recurrence risk and poorer prognosis, even in early-stage LUAD, especially stage I disease [27]. This underscores the clinical relevance of mutation detection in small-sized nodules, where early risk stratification may support individualized management. In this context, radiomics offers a promising non-invasive approach for genomic prediction. Several prior studies have reported encouraging results. For instance, Li et al. [18] developed a deep learning model using CT scans from 844 patients, automatically learning features from 14,926 images, and achieved AUCs of 0.85 and 0.81 in the training and validation cohorts, respectively—significantly outperforming both radiomics and clinical models. In comparison, Zhao et al. [28] used non-contrast CT scans from 404 NSCLC patients, extracting 234 radiomics features, and constructed a model with AUCs of 0.762 and 0.775; integrating clinical variables further improved the AUC to 0.818, highlighting the benefit of multimodal input. the relatively high AUCs reported in some studies may be partially attributed to the inclusion of potentially confounding variables such as tumor location (e.g. central vs. peripheral), which are intrinsically associated with histological subtypes—central tumors are more likely to be squamous cell carcinoma and EGFR-wild type—thereby introducing bias in mutation prediction. Similarly, Liu et al. [29] analyzed 298 peripheral lung adenocarcinoma cases, selecting 59 independent features from an initial set of 219, and reported an AUC of 0.647 for the radiomics model alone, which improved to 0.709 when combined with clinical predictors such as smoking status and pathologic grade. More recently, Cheng et al. [19] focused on 636 patients with ground-glass opacity (GGO)-featured lung adenocarcinoma, extracting 1,476 features and selecting 102 for model construction. Their radiomics model achieved AUCs of 0.838, 0.822, and 0.803 across training, internal, and external cohorts, and notably improved EGFR-TKI treatment response in a clinical validation cohort (response rate: 25.9% vs. 53.8%, *p* = 0.006). Compared with these studies, our model achieves comparable predictive performance using only 15 features and non-contrast CT, reducing feature redundancy and improving generalizability.

In addition to feature parsimony, our model design addresses multiple limitations observed in previous studies. First, we deliberately avoided potential confounding variables such as tumor location (central vs. peripheral), which are inherently linked to histological subtypes and may bias mutation prediction. Second, unlike deep learning models that often lack transparency, our machine learning–based approach leverages SHAP analysis to enable visual interpretation of feature contributions, promoting clinical adoption. Third, we retained CTR as a continuous variable rather than dichotomizing it using arbitrary thresholds, preserving the full predictive spectrum of this feature and improving the model’s sensitivity to subtle tumor characteristics.

Furthermore, analysis of SHAP values revealed that CTR was the most influential feature in the model, followed by two first-order variance features extracted from wavelet‑transformed and original images. First‑order variance features reflect intratumoral heterogeneity, a known imaging surrogate that has been associated with EGFR mutation status in lung adenocarcinoma; higher homogeneity (i.e. lower variance) has been correlated with EGFR‑mutant tumors [30,31]. Clinical variables such as smoking status and sex also ranked highly, which aligns with epidemiologic trends showing EGFR mutations are more prevalent in non-smoking females [32]. Interestingly, texture-based features derived from GLCM, GLSZM, or GLDM contributed only marginally to model output. As shown in the SHAP analysis (Figure 4B), first-order variance features (R1, R6) had markedly higher contributions compared to texture-based features such as GLCM (R3, R7), GLSZM (R12), and GLDM (R11), supporting the interpretation that first-order statistical descriptors capture more robust signals in small non-contrast CT nodules. Taken together, SHAP-based feature attribution not only reveals the model’s decision logic but also underscores the biological and clinical plausibility of feature choices, reinforcing the model’s interpretability and potential for clinical integration.

Building on these findings, our results further suggest that elevated CTR and increased textural homogeneity are closely associated with EGFR mutation status, and these imaging phenotypes may represent not merely statistical differences but macroscopic reflections of underlying biological mechanisms. Multiple studies have demonstrated that omics-based signatures, including radiomic [33,34], proteogenomic [35], DNA damage repair-related [36,37], dynamic network [38], and mitochondrial metabolism [39,40] signatures, can effectively predict variations in the tumor immune microenvironment, immunotherapy response, and patient prognosis [41]. Collectively, this evidence highlights the strong association between omics features and immune states. Similarly, radiomics studies have shown that CT-derived features can predict PD-L1 expression [42], immune cell infiltration [43], and immunotherapy outcomes [43,44], suggesting their potential role as imaging surrogates of the tumor immune microenvironment. Therefore, it is reasonable to hypothesize that the elevated CTR and textural homogeneity observed in our study may correspond to the typical immunological phenotype of EGFR-mutant LUAD—namely, an ‘immune-cold’ state characterized by insufficient T-cell infiltration, suppressed cytokine activity, and metabolic reprogramming [45–47]. In future work, we will conduct further investigations into the roles of CTR and radiomics-derived textural features in shaping the tumor immune microenvironment, thereby providing stronger theoretical and empirical support for the hypotheses proposed in this study.

Beyond the overall mutation status, the prediction of EGFR mutation subtypes such as exon 19 deletions, L858R, and T790M is of considerable clinical relevance [48,49]. These subtypes not only differ in their biological behavior but also exhibit distinct sensitivities and resistance patterns to EGFR-TKIs [50,51]. For instance, exon 19 deletions and L858R mutations confer favorable responses to first- and third-generation TKIs, whereas the emergence of T790M is a well-known mechanism of acquired resistance. Therefore, a non-invasive imaging-based tool capable of differentiating EGFR mutation subtypes would provide further value in guiding treatment selection and monitoring therapeutic resistance. Although the sample size of the present study did not allow reliable subtype-level modeling, our findings establish a methodological foundation, highlighting the potential feasibility of radiomics-driven subtype prediction. With larger datasets and external validation, future research may extend this approach to refine molecular stratification and enable personalized therapeutic strategies for LUAD patients.

Despite its strengths, our study has several limitations. First, the retrospective design may introduce selection bias, particularly due to the exclusion of cases with incomplete clinical or imaging data. Second, although internal validation was conducted using a split-sample approach, external validation in independent, multicenter cohorts is needed to confirm generalizability. Third, CT imaging protocols may vary across institutions could affect feature reproducibility. Although standard preprocessing was applied, future work should explore harmonization strategies. In addition, the absence of temporal validation and external datasets under heterogeneous scanner and reconstruction conditions may limit generalizability. Finally, the sensitivity and specificity of our model were moderate, likely due to the limited imaging information in small non-contrast nodules. Besides, we did not adopt fully nested cross-validation because of an independent hold-out test cohort; to mitigate optimism, we used 1,000-bootstrap resampling and report calibration metrics. Future work will consider nested CV in multi-center settings.

## Conclusion

This study presents a noninvasive, interpretable random forest model that integrates CT radiomics, CTR, and selected clinical variables to predict EGFR mutation status in small (≤ 3 cm) lung adenocarcinoma nodules. The combined model outperformed radiomics-only and clinical-only approaches, with CTR identified as the most influential predictor. Model interpretability analysis supports its potential as a practical decision-support tool when tissue genotyping is unavailable, offering guidance for individualized management in early-stage lung cancer. Prospective multicenter studies are needed to confirm its generalizability and promote clinical adoption.

## Supplementary Material

supplementary table.docx

FigS1.jpg

## Data Availability

The data that support the findings of this study are available from the corresponding author uponreasonable request.
