# Supplementary material for: Interpretable machine learning model integrating CT radiomics, CTR, and clinical features for EGFR mutation prediction in ≤3 cm lung adenocarcinoma nodules
Source: Ann Med. 2025 Dec 19;57(1):2607160. doi: 10.1080/07853890.2025.2607160 (PMC12720629; doi:10.1080/07853890.2025.2607160)
Supplement: supplementary table.docx [file IANN_A_2607160_SM5415.docx]

**Supplementary** **Table S1. The final selected Radiomics features For Radiomics model**

| Feature ID | Feature Name |
| --- | --- |
| R1 | wavelet-LLL_firstorder_Variance |
| R2 | wavelet-LHH_firstorder_Skewness |
| R3 | log-sigma-1-0-mm-3D_glcm_Imc2 |
| R4 | wavelet-LLH_glcm_Correlation |
| R5 | log-sigma-5-0-mm-3D_firstorder_Median |
| R6 | original_firstorder_Variance |
| R7 | original_glcm_MaximumProbability |
| R8 | wavelet-LLL_glrlm_LowGrayLevelRunEmphasis |
| R9 | log-sigma-3-0-mm-3D_firstorder_90Percentile |
| R10 | log-sigma-5-0-mm-3D_firstorder_Mean |
| R11 | wavelet-LLL_gldm_LowGrayLevelEmphasis |
| R12 | wavelet-LLL_glszm_LowGrayLevelZoneEmphasis |

**Supplementary Table S2. Performance metrics of the clinical logistic regression model in the training and testing cohorts**

| model_name | AUC | 95% CI | Accuracy | Sensitivity | Specificity | PPV | NPV | Task |
| --- | --- | --- | --- | --- | --- | --- | --- | --- |
| LogisticRegression | 0.61 | 0.55 - 0.66 | 0.62 | 0.88 | 0.25 | 0.63 | 0.58 | Training set |
| LogisticRegression | 0.60 | 0.49 - 0.71 | 0.59 | 0.86 | 0.21 | 0.61 | 0.53 | Test set |

AUC, area under the curve; CI, confidence interval; PPV, positive predictive value; NPV, negative predictive value; SVM, Support vector machine; KNN, K-Nearest Neighbors; XGBoost, Extreme gradient boosting; LightGBM, Light gradient boosting machine

**Supplementary Table S3. Performance metrics of seven radiomics-based machine learning models in the training and testing cohorts**

| model_name | AUC | 95% CI | Accuracy | Sensitivity | Specificity | PPV | NPV | Task |
| --- | --- | --- | --- | --- | --- | --- | --- | --- |
| SVM | 0.73 | 0.67 - 0.78 | 0.72 | 0.89 | 0.49 | 0.71 | 0.74 | Training set |
| SVM | 0.68 | 0.56 - 0.78 | 0.62 | 0.90 | 0.24 | 0.62 | 0.63 | Test set |
| Logistic Regression | 0.71 | 0.65 - 0.76 | 0.70 | 0.87 | 0.45 | 0.69 | 0.71 | Training set |
| Logistic Regression | 0.67 | 0.56 - 0.78 | 0.63 | 0.90 | 0.26 | 0.63 | 0.65 | Test set |
| KNN | 0.68 | 0.62 - 0.74 | 0.66 | 0.81 | 0.43 | 0.67 | 0.61 | Training set |
| KNN | 0.63 | 0.51 - 0.73 | 0.64 | 0.89 | 0.31 | 0.64 | 0.65 | Test set |
| Decision Tree | 0.58 | 0.52 - 0.63 | 0.59 | 0.65 | 0.50 | 0.65 | 0.50 | Training set |
| Decision Tree | 0.58 | 0.47 - 0.68 | 0.62 | 0.73 | 0.48 | 0.66 | 0.56 | Test set |
| Random Forest | 0.68 | 0.63 - 0.74 | 0.65 | 0.76 | 0.49 | 0.68 | 0.58 | Training set |
| Random Forest | 0.69 | 0.58 - 0.79 | 0.65 | 0.90 | 0.31 | 0.65 | 0.68 | Test set |
| XGBoost | 0.63 | 0.57 - 0.69 | 0.63 | 0.73 | 0.48 | 0.67 | 0.55 | Training set |
| XGBoost | 0.65 | 0.55 - 0.75 | 0.60 | 0.78 | 0.36 | 0.63 | 0.54 | Test set |
| LightGBM | 0.65 | 0.59 - 0.70 | 0.63 | 0.75 | 0.46 | 0.67 | 0.56 | Training set |
| LightGBM | 0.65 | 0.54 - 0.76 | 0.60 | 0.78 | 0.36 | 0.63 | 0.54 | Test set |

AUC, area under the curve; CI, confidence interval; PPV, positive predictive value; NPV, negative predictive value; SVM, Support vector machine; KNN, K-Nearest Neighbors; XGBoost, Extreme gradient boosting; LightGBM, Light gradient boosting machine

**Supplementary Table S4. Performance metrics of the combined clinical–radiomics model in the training and testing cohorts**

| model_name | AUC | 95% CI | Accuracy | Sensitivity | Specificity | PPV | NPV | Task |
| --- | --- | --- | --- | --- | --- | --- | --- | --- |
| SVM | 0.79 | 0.74 - 0.83 | 0.71 | 0.85 | 0.51 | 0.71 | 0.72 | Training set |
| SVM | 0.71 | 0.61 - 0.82 | 0.67 | 0.86 | 0.40 | 0.67 | 0.68 | Test set |
| LogisticRegression | 0.72 | 0.67 - 0.77 | 0.69 | 0.83 | 0.48 | 0.70 | 0.67 | Training set |
| LogisticRegression | 0.69 | 0.58 - 0.79 | 0.64 | 0.88 | 0.31 | 0.64 | 0.65 | Test set |
| KNN | 0.73 | 0.68 - 0.78 | 0.67 | 0.85 | 0.40 | 0.67 | 0.66 | Training set |
| KNN | 0.69 | 0.59 - 0.79 | 0.65 | 0.85 | 0.38 | 0.66 | 0.64 | Test set |
| DecisionTree | 0.64 | 0.58 - 0.69 | 0.65 | 0.73 | 0.54 | 0.69 | 0.59 | Training set |
| DecisionTree | 0.60 | 0.50 - 0.70 | 0.61 | 0.71 | 0.48 | 0.66 | 0.54 | Test set |
| RandomForest | 0.74 | 0.69 - 0.79 | 0.70 | 0.80 | 0.55 | 0.72 | 0.66 | Training set |
| RandomForest | 0.76 | 0.66 - 0.85 | 0.69 | 0.90 | 0.40 | 0.68 | 0.74 | Test set |
| XGBoost | 0.72 | 0.66 - 0.77 | 0.68 | 0.76 | 0.58 | 0.72 | 0.62 | Training set |
| XGBoost | 0.70 | 0.60 - 0.81 | 0.68 | 0.85 | 0.45 | 0.68 | 0.68 | Test set |
| LightGBM | 0.73 | 0.67 - 0.78 | 0.67 | 0.74 | 0.57 | 0.72 | 0.61 | Training set |
| LightGBM | 0.70 | 0.59 - 0.80 | 0.75 | 0.88 | 0.57 | 0.74 | 0.77 | Test set |

AUC, area under the curve; CI, confidence interval; PPV, positive predictive value; NPV, negative predictive value; SVM, Support vector machine; KNN, K-Nearest Neighbors; XGBoost, Extreme gradient boosting; LightGBM, Light gradient boosting machine

| Model | N | Prevalence | ROC AUC | Brier | Scaled Brier | Calib intercept | Calib slope |
| --- | --- | --- | --- | --- | --- | --- | --- |
| Cli | 98 | 0.58 | 0.60 | 0.24 | 0.03 | -0.08 | 1.0747894616667584 |
| Rad | 98 | 0.58 | 0.69 | 0.22 | 0.09 | -0.07 | 0.8389767217600735 |
| Com | 98 | 0.58 | 0.76 | 0.20 | 0.19 | -0.36 | 1.3838290566907512 |

**Supplementary Table S5. Calibration performance of the clinical, radiomics, and combined models in the independent test cohort**

AUC, area under the curve.
