# Supplementary figures and images for: Interpretable machine learning model integrating CT radiomics, CTR, and clinical features for EGFR mutation prediction in ≤3 cm lung adenocarcinoma nodules
Source: Ann Med. 2025 Dec 19;57(1):2607160. doi: 10.1080/07853890.2025.2607160 (PMC12720629; doi:10.1080/07853890.2025.2607160)

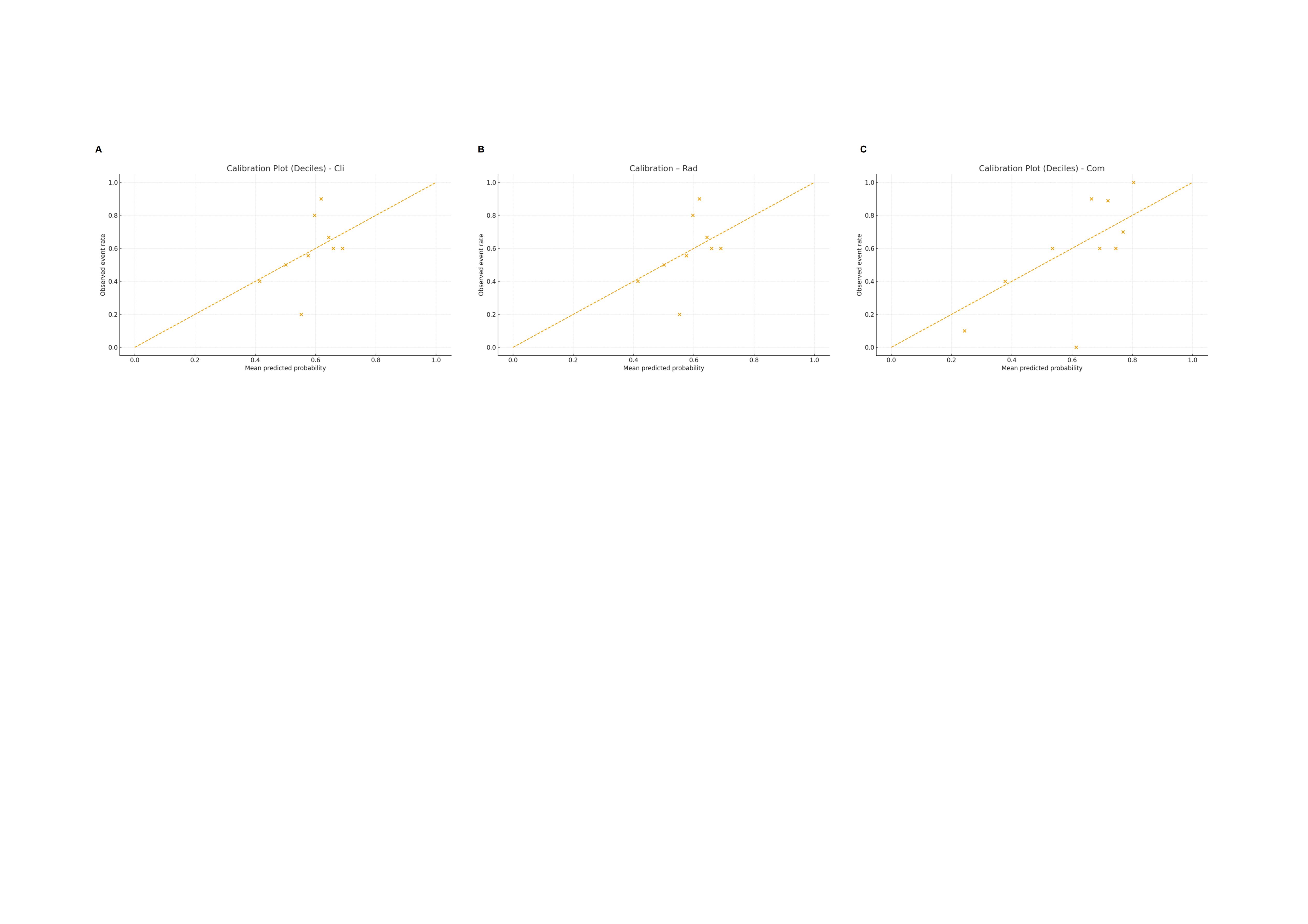

Supplement: FigS1.jpg [file IANN_A_2607160_SM5414.jpg]
